# Elephantiasis Nostras Verrucosa in a Patient With Lymphedema Tarda

**DOI:** 10.7759/cureus.56850

**Published:** 2024-03-24

**Authors:** Sharwari Jaiswal, Bhushan Madke, Adarshlata Singh, Nitya Vangala, Shivani D Jangid

**Affiliations:** 1 Dermatology, Jawaharlal Nehru Medical College, Datta Meghe Institute of Higher Education & Research, Wardha, IND; 2 Dermatology, Mahavir Institute of Medical Sciences, Vikarabad, IND

**Keywords:** comprehensive management, diagnostic challenges, multidisciplinary approach, chronic lymphatic disorders, elephantiasis nostras verrucosa, lymphedema tarda

## Abstract

This case report presents a rare and complex clinical scenario of a 42-year-old male diagnosed with elephantiasis nostras verrucosa in the context of lymphedema tarda. The patient's seven-year history of insidious and progressively worsening swelling over the left lower limb, inguino-scrotal region, and left upper limb posed diagnostic challenges, leading to a multidisciplinary evaluation. Clinical examination, imaging studies, and laboratory investigations were integral in confirming the diagnosis. The manifestation of elephantiasis nostras verrucosa, characterized by extensive hyperkeratosis, added a unique dimension to the clinical presentation. A comprehensive treatment approach involving nutritional supplementation and pharmacological interventions was initiated to address the multifaceted aspects of lymphatic dysfunction. This case underscores the importance of a collaborative and holistic approach to managing complex lymphatic disorders, contributing valuable insights to the medical literature.

## Introduction

Lymphedema tarda, characterized by a delayed onset of lymphedema typically occurring after age 35, is a rare and often challenging condition to manage [1]. It is associated with impaired lymphatic drainage and accumulating protein-rich fluid in the interstitial tissues [2]. One of the unusual manifestations of chronic lymphedema is elephantiasis nostras verrucosa, a condition marked by massive, verrucous hyperkeratosis resulting from chronic lymphatic obstruction [3]. Chronic lymphedema can arise from various etiologies, including surgical procedures, trauma, infection, or congenital malformations [4]. In the case of lymphedema tarda, the delayed onset and insidious progression pose diagnostic challenges, often necessitating a multidisciplinary approach for accurate evaluation and management [5].

Elephantiasis nostras verrucosa, though rare, has been documented in the literature as a consequence of long-standing lymphatic stasis. It is characterized by the hypertrophy of the epidermis, leading to a thickened and verrucous appearance, particularly in the affected extremities [3]. The rarity of elephantiasis nostras verrucosa and lymphedema tarda makes this case notable. The complex interplay of lymphatic dysfunction, chronic edema, and the development of verrucous hyperkeratosis necessitates a thorough understanding of the underlying pathophysiology for effective management.

Diagnosing these conditions often involves a combination of clinical examination, imaging studies, and laboratory investigations. Ultrasound and Doppler studies are instrumental in assessing lymphatic anatomy and function, while dermatological examination aids in recognizing characteristic skin changes [6]. Managing lymphedema tarda and elephantiasis nostras verrucosa requires a comprehensive approach, addressing the underlying lymphatic dysfunction and associated complications. Pharmacological interventions, such as Hetrazan, aim to improve lymphatic flow, while nutritional supplementation may be necessary to address deficiencies contributing to the condition's progression [7]. This case report contributes to the existing body of literature by presenting a comprehensive evaluation and management strategy for a patient with elephantiasis nostras verrucosa in the setting of lymphedema tarda, highlighting the importance of collaboration among healthcare professionals in optimizing patient outcomes.

## Case presentation

**Figure 1:**
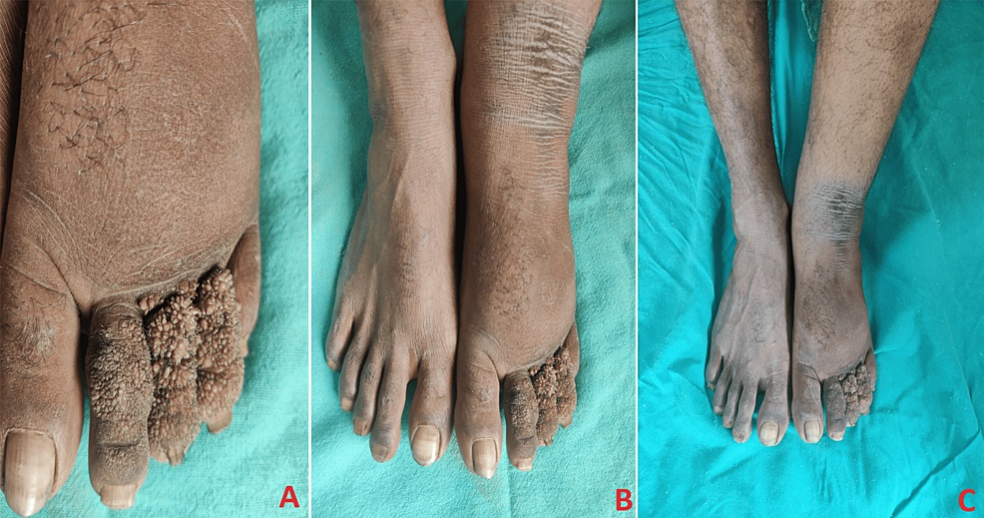
(A, B, C) The lymphovenous anastomosis revealed evidence of grade I/II lymphedema in the left lower limb and mild delayed transit of tracer through a few dilated lymphatic channels in the left upper limb

A 42-year-old male presented to the outpatient department of a tertiary care hospital with a complaint of swelling in the left lower limb for seven years, swelling in the inguino-scrotal region for five years, and swelling in the left upper limb for two years. The patient complained about the insidious onset of swelling seven years ago, gradually progressing to involve the left lower limb. The patient had a history of hospitalization with similar complaints and was advised to undergo lymphovenous anastomosis. The lymphovenous anastomosis revealed evidence of grade I/II lymphedema in the left lower limb and mild delayed transit of tracer through a few dilated lymphatic channels in the left upper limb (Figure 1). The delayed visualization of left axillary nodes also suggested grade II lymphedema in the left upper limb. The patient also reported a progressive swelling in the inguino-scrotal region involving the scrotum and penis.

During the local examination of the left lower limb, dry skin, swelling, and hyperkeratosis over the toes of the left foot were observed. Dilated veins, local rise in temperature, tenderness over the left ankle, and pitting-type edema were noted. Arterial pulses in the dorsalis pedis, posterior tibial, and popliteal were not palpable. Examination of the left upper limb revealed normal skin without dilated veins. Pitting edema was noted on the left hand, and peripheral pulse on the left upper limb was not palpable. Blood tests, including complete blood count, renal function test, and liver function test, were within the normal range. Ultrasonography of the inguino-scrotal region revealed a heterogeneous texture of bilateral testes, with thickening of the scrotal wall. Enlarged lymph nodes were noted in the bilateral inguinal region, with the largest measuring 11×6 mm on the left side.

**Figure 2:**
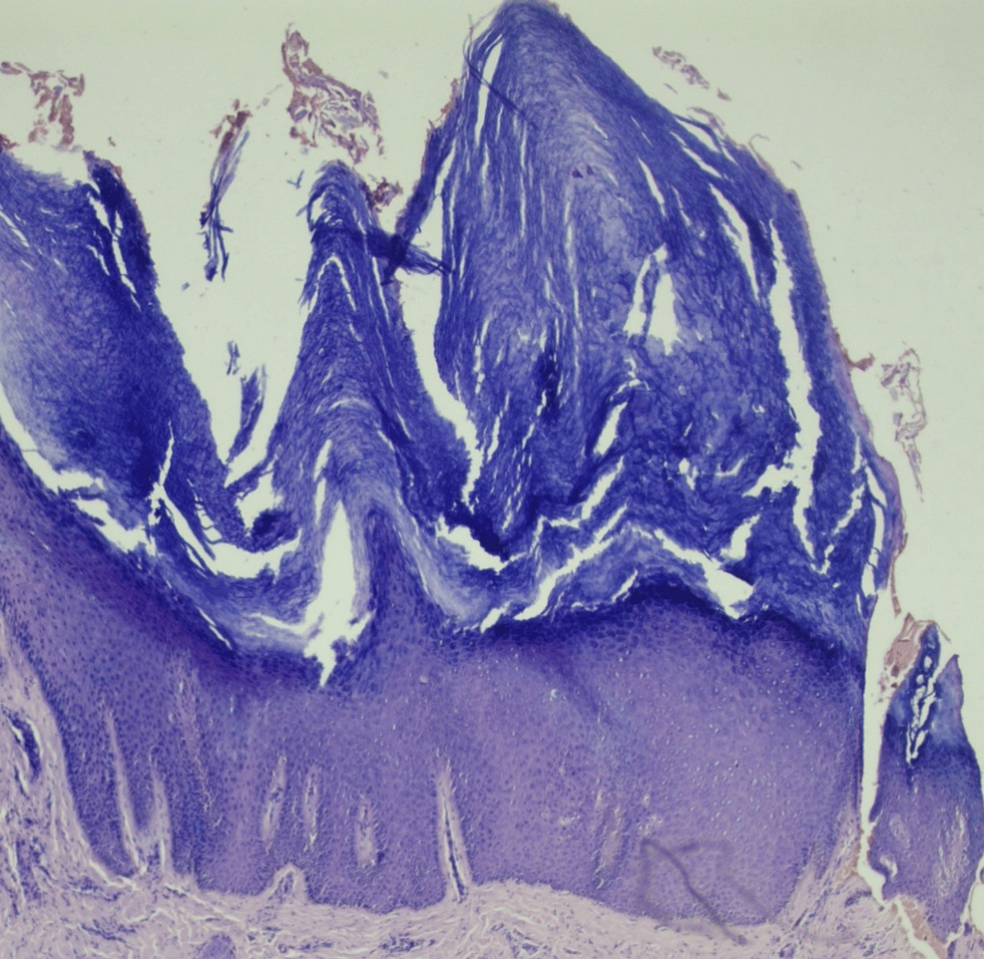
Hyperkeratosis, papillomatosis, and pseudoepitheliomatous hyperplasia of the epidermis and dermal fibrosis

A duplex study of bilateral lower limbs (arterial and venous) showed dilation of the great saphenous vein throughout its course, measuring 4.3 mm in the left lower limb with multiple varicosities. The small saphenous vein measured 2.2 mm, displaying superficial varicosities along its course in the left lower limb. Histologic examination demonstrates hyperkeratosis, papillomatosis, and pseudoepitheliomatous hyperplasia of the epidermis and dermal fibrosis. Multiple varicose veins were observed after a detailed examination of the lower foot for hyperkeratotic lesions over the second, third, and fourth patients with elephantiasis nostras verrucosa with lymphedema tarda (Figure 2). Treatment was initiated with multivitamins, trypsin-chymotrypsin combination, pantoprazole, diethylcarbamazine, and vitamin C under the doctor's supervision.

## Discussion

The presented case of a 42-year-old male with elephantiasis nostras verrucosa in the context of lymphedema tarda highlights the complexities associated with the delayed onset and chronic progression of lymphatic disorders. Lymphedema tarda is a rare condition characterized by the late onset of lymphedema, typically manifesting after age 35 [8]. The delayed presentation, in this case, underscores the importance of considering lymphatic dysfunction in patients presenting with chronic edema, even in the absence of acute triggers. The insidious progression of lymphedema tarda may lead to complications such as fibrosis, impaired immune function, and, as observed in this case, the development of elephantiasis nostras verrucosa [9].

Elephantiasis nostras verrucosa is an unusual manifestation of chronic lymphedema, characterized by massive hyperkeratosis and verrucous skin changes resulting from prolonged lymphatic stasis [10]. The development of this condition is often linked to long-standing lymphatic obstruction, leading to altered tissue architecture and chronic inflammation. In the presented case, the extensive hyperkeratosis over the toes and lower limb and the involvement of the inguino-scrotal region align with typical features of elephantiasis nostras verrucosa. Diagnostic evaluation of lymphatic disorders involves a combination of clinical examination, imaging studies, and laboratory investigations. Ultrasonography and Doppler studies help assess lymphatic anatomy and function, while dermatological examination aids in recognizing characteristic skin changes [2]. In our case, the diagnostic journey included ultrasonography for inguino-scrotal evaluation and duplex colour Doppler study for the lower limbs, facilitating a comprehensive understanding of the extent of lymphatic involvement.

Multidisciplinary management is crucial for optimizing outcomes in patients with complex lymphatic disorders. In this case, the patient was referred to a dermatologist for a detailed assessment of skin changes. The treatment regimen included a combination of medications addressing nutritional deficiencies, inflammation, and lymphatic dysfunction, reflecting a holistic approach to patient care. Hetrazan, an antifilarial agent, was included in the treatment plan to improve lymphatic flow and reduce the progression of lymphedema [11]. Nutritional supplementation with Supradyn and Limcee aimed to address potential deficiencies contributing to the chronicity of the condition. Chymoral Forte was prescribed to manage inflammation, and pantoprazole was included for gastroprotection.

## Conclusions

This case report sheds light on the intricate nature of lymphatic disorders, exemplified by a 42-year-old male presenting with elephantiasis nostras verrucosa in the setting of lymphedema tarda. The delayed onset of lymphedema tarda, a rare condition, underscores the diagnostic challenges associated with chronic lymphatic dysfunction. The manifestation of elephantiasis nostras verrucosa further adds complexity, emphasizing the importance of a multidisciplinary approach for accurate diagnosis and comprehensive management. The thorough clinical examination, imaging studies, and laboratory investigations played pivotal roles in unraveling the intricacies of this case. The treatment regimen, encompassing pharmacological interventions and nutritional supplementation, exemplifies the need for a holistic approach to address the diverse facets of lymphatic disorders. This case contributes to the existing literature by emphasizing the significance of collaboration among healthcare professionals in navigating the complexities of lymphatic disorders, ensuring a comprehensive and tailored approach for improved patient outcomes.
